# Progress and Prospects of Research on Microhabitat Scale Characterization and Utilization in Chinese Karst Area

**DOI:** 10.1002/ece3.72646

**Published:** 2025-12-12

**Authors:** Hui Huang, Yanghua Yu, Shunsong Yang, Yun Yang, Yurong Fu

**Affiliations:** ^1^ School of Karst Science, State Engineering Technology Institute for Karst Decertification Control Guizhou Normal University Guiyang China; ^2^ School of Geography and Environmental Sciences Guizhou Normal University Guiyang China

**Keywords:** bibliometrics, connectivity, ecological restoration, heterogeneity, resource allocation, surface‐subsurface coupling

## Abstract

Karst microhabitats, characterized by their complex structures and unique functions, serve as critical habitats and resource reservoirs for biodiversity. However, systematic research in this area remains limited. This study conducts a systematic literature review based on publications from the China National Knowledge Infrastructure (CNKI) and Web of Science (WOS) databases, with a focus on karst microhabitats in China. The objectives are to synthesize domestic research progress and compare it with international studies to enhance the understanding of microhabitats. The findings reveal that: (i) the number of publications on this topic has generally increased, with CNKI literature predominantly centered on karst and microhabitat entities (47% of the total), whereas WOS publications place greater emphasis on biodiversity within microhabitats (24%); (ii) key advances and landmark findings from studies conducted at different scales in Chinese karst regions are highlighted, along with a summary of critical scientific issues; and (iii) representative techniques applicable to karst microhabitats in China are evaluated, drawing on international research experience and their adaptive outcomes. Moving forward, research on karst microhabitats in China should prioritize quantitative analysis of subsurface spatial characteristics and integrate interdisciplinary approaches to elucidate resource allocation mechanisms.

## Graphical abstract

Karst regions exhibit ecologically fragile environments but host a rich diversity of microhabitats across spatial scales. Vegetation productivity and carbon sequestration patterns are intricately governed by subterranean microhabitat conditions. Microhabitat‐based technologies demonstrate transformative potential in reconciling human livelihoods with ecological restoration.

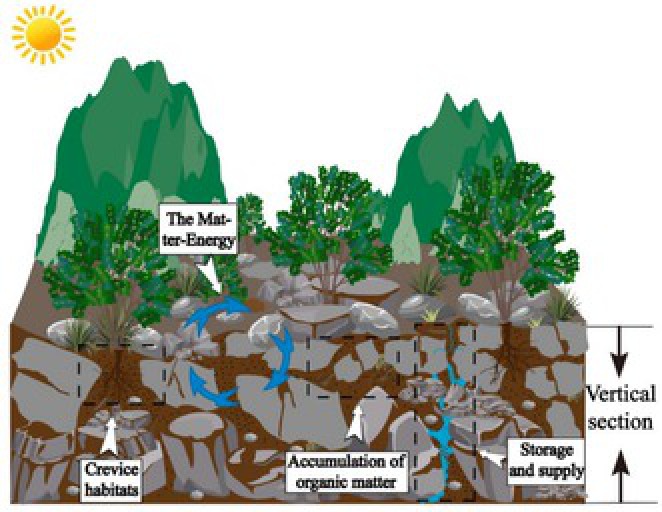

## 1 Introduction

Karst landforms cover approximately 15% of the global land area and are distributed across multiple regions, including the Dinaric Alps in Bosnia and Herzegovina, the French Massif Central, the Ural Mountains in Russia, southern Australia, the mid‐eastern United States, the Greater Antilles, north‐central Vietnam, and southwestern China (Goldscheider et al. 2020; Lipar et al. 2024). Among these, the karst area in Southwest China is the world's largest and most continuous expanse of its kind. It features typical karst landscapes, characterized by peak clusters and pinnacles, earning it the reputation as a natural karst museum (Zou et al. 2025). Long‐term dissolution and deposition processes have not only shaped the geochemical cycling of carbonate rocks but also created diverse surface and subsurface landscapes (Ford and Williams 2007), making this region an ideal system for studying subsurface–surface material transport and carbon sink dynamics (Zeng et al. 2025). In this context, karst microhabitats defined as natural units influenced by soil depth, light availability, slope position, and other micro‐environmental factors (Gao et al. 2022), serve dual ecological roles as both cradles of life and species refugia (Bátori et al. 2023). They constitute the structural and functional foundation of regional ecosystems. Nevertheless, microhabitats exhibit considerable spatial heterogeneity, and their formation mechanisms, resource distribution patterns, and ecological functions remain poorly understood in a systematic framework.

Karst microhabitats can be divided into surface and subsurface types, both of which play vital roles in maintaining biodiversity. For instance, limestone fissure habitats facilitate deep root penetration, support the establishment of a greater number of species, and promote the development of complex aboveground vegetation structures (Liu et al. 2019). These microhabitats also alter relationships among leaf traits (Touré and Ge 2014), leading to more complex and integrated trait networks. However, due to the inherent fragility of karst ecosystems and combined pressures from natural and anthropogenic disturbances, issues such as soil loss, vegetation degradation, and rocky desertification are widespread (Xiong et al. 2025). In response, the Chinese government has initiated a range of ecological restoration programs (Yu and Pu 2025). A core strategy involves the rational use of microhabitats for vegetation recovery, which enhances restoration efficiency, promotes agroforestry development, and harmonizes ecological, economic, and social benefits (Gupta et al. 2020; Li et al. 2025). Therefore, prior to utilizing microhabitats for accelerating vegetation restoration, it is essential to clarify how plant communities respond to different microhabitat conditions. Based on regional habitat characteristics, scientifically assessing the carrying capacity of microhabitats and coordinating the functional synergy between surface and subsurface microhabitats in material and energy flows will benefit both ecological recovery and societal development.

Current research on karst microhabitats in China has primarily focused on surface environments (Liao et al. 2010; Peng, Tang, et al. 2023; Peng, Xu, et al. 2023), with an emphasis on plant growth and soil properties. Although subsurface microhabitats are difficult to access and have long been treated as a “black box,” recent studies have yielded substantial findings regarding their spatial structure, soil characteristics, and plant adaptations (Fu et al. 2022). Nevertheless, effective detection techniques for subsurface microhabitats and a systematic understanding of aboveground–belowground linkage mechanisms are still lacking. To address these gaps, this paper aims to systematically review research progress on karst microhabitats in China. By synthesizing the characteristics, utilization techniques, and key scientific questions related to microhabitats across different scales, we explore their roles in resource allocation and ecological regulation. Through this effort, we seek to enhance the understanding of karst microhabitats in this region and provide a reference for research in similar karst environments worldwide.

## 2 Data Sources and Analyses

### 2.1 Data Sources and Selection

We followed the systematic literature review methodology, drawing on its logical and systematic characteristics to identify primary research directions through a transparent and bounded research path (van Dinter et al. 2021). To ensure the comparability of Chinese and English literature datasets in terms of temporal scope and to investigate the research evolution in the field of karst microhabitats both domestically and internationally, we searched the Core Collection of the Chinese National Knowledge Infrastructure (CNKI) database and the Web of Science (WOS) database from August 1989 to August 2024. In both databases, we limited the document type to peer‐reviewed research articles to ensure consistency in publication type. For the CNKI database, we used the advanced search function and set the topic as “karst microhabitat”, or “karst habitat”, with the language restricted to Chinese and the document type set to “Academic Journal”. This initial search yielded 276 publications. After manually excluding reviews, conference papers, and duplicates, 203 valid research articles were obtained. For the WOS database, the Web of Science Core Collection was searched using the topic terms “karst microhabitat”, or “karst habitat”, with the language limited to English and the document type strictly defined as “Article”. This initial search retrieved 1753 English publications. Following the same manual screening procedure, 1216 valid articles were retained for analysis.

### 2.2 Research Methodology

This study utilized CiteSpace 6.3 R1 (https://citespace.podia.com/), a Java‐based software, for data processing and literature visualization to track and analyze microhabitat research (Azam et al. 2021). The software enables analysis on the basis of authorship, keyword co‐occurrence, clustering, and other visual representations, making research findings more intuitive, salient, and persuasive (Yohannes et al. 2024). Known for its user‐friendly interface and ease of use, CiteSpace provides robust capabilities for visually interpreting and exploring knowledge structures. It addresses the limitations of other tools, such as VOSviewer's lack of clustering and temporal analysis, HistCite's inability to deeply analyze complex literature relationships and network structures, and RefViz's challenges in conducting comprehensive analyses. As a result, it has gained widespread acceptance among researchers globally (Kumar et al. 2023; Geng et al. 2024).

### 2.3 Analysis of the Results

#### 2.3.1 Analysis of Trends in the Development of Literature

**FIGURE 1:**
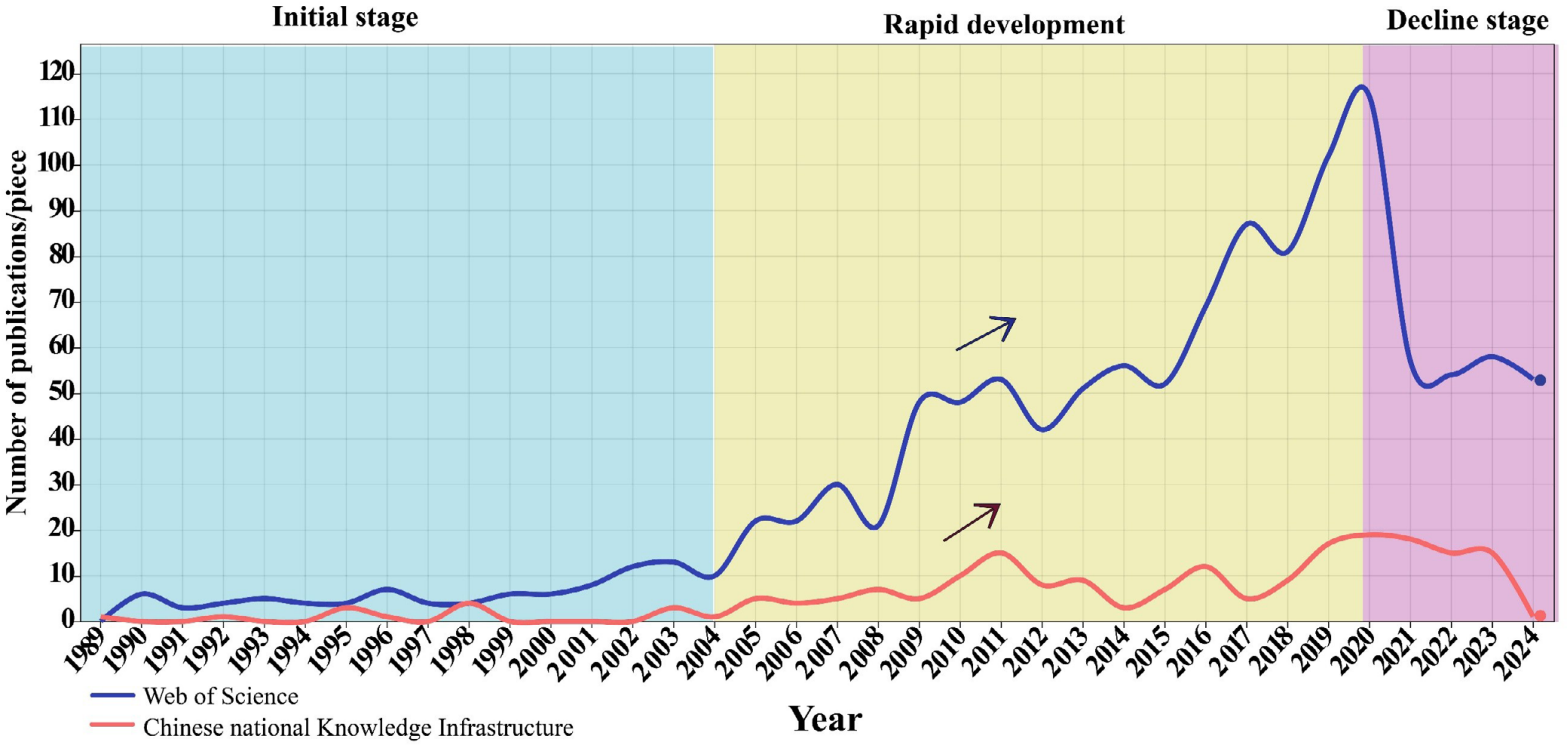
Annual trends in the publication of karst microhabitats literature. (The data in the FIGURE is based on the annual number of research articles retrieved from the CNKI and WOS databases that meet our inclusion criteria).

Figure 1 illustrates the annual number of relevant research articles published between 1989 and 2024 in the CNKI and WOS databases. Based on publication trends, the research progression can be divided into three stages. Prior to 2004 constituted the initial stage, with relatively stable output focusing on climatic characteristics of microhabitats and vegetation restoration in mountainous habitats (Zhang et al. 1995; Jonášová and Prach 2004). From 2004 to 2020, the field entered a growth stage, marked by increased activity and studies concentrating on vegetation traits, soil properties, subterranean caves, and hydrological microhabitats (Peng, Dai, et al. 2020; Peng, Song, et al. 2020; Morrissey et al. 2020). After 2020, the COVID‐19 pandemic led to a decline in overall scientific output, resulting in a reduction in annual publications from their peak. Research during this period emphasized plant adaptation strategies to microhabitats (Chen et al. 2020; Yang et al. 2024).

#### 2.3.2 Analysis of Authors of Publications

**FIGURE 2:**
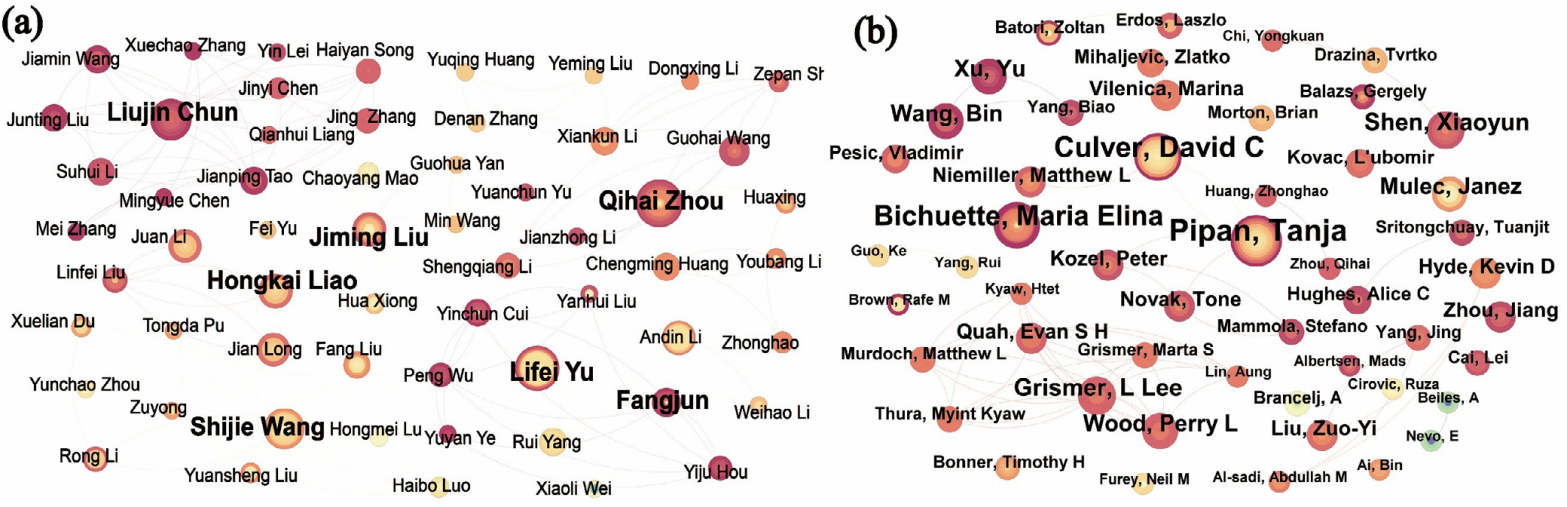
(a) Author co‐occurrence map of Chinese National Knowledgeable Infrastructure literature; (b) Author co‐occurrence map of Web of Science literature; larger nodes represent authors with a higher number of publications.

Through analyzing the number of publications and co‐authorship networks, we constructed a co‐authorship map (Figure 2), which reveals notable regional differences in karst microhabitat research. In the CNKI database, core authors such as Zhou Qihai, Yu Lifei, and Wang Shijie have focused on species diversity, forest ecology, and biogeochemistry, respectively, forming the backbone of domestic research in this field. Their studies are largely concentrated on application‐oriented topics such as rocky desertification control, vegetation restoration, and ecosystem service assessment, indicating that Chinese scholars often align their work with national ecological engineering priorities (Chen, Zhang, et al. 2024; Chen, Cheng, et al. 2024). In contrast, core authors in the WOS database, including Tanja Pipan, Culver David C, and Bichuette Maria Elina, place greater emphasis on fundamental scientific questions such as cave ecology, subterranean biodiversity, and organismal adaptation, reflecting a stronger international focus on elucidating environment–species interaction mechanisms (Frei et al. 2023).

These differences highlight the need to integrate global theoretical advances with localized restoration practices in China. Furthermore, while domestic studies pay limited attention to subsurface processes due to habitat complexity, international research offers less insight into ecohydrological processes in surface microhabitats. Enhanced collaboration between Chinese and international researchers is essential to bridge the gap between basic research and applied goals.

#### 2.3.3 Keyword Analysis

##### 2.3.3.1 Keyword Co‐Occurrence Analysis

Keywords are a highly condensed representation of an article's theme and serve as its effective key information. Analyzing high‐frequency keywords in a specific field helps uncover relevant research hotspots in that domain (Bamisile et al. 2023). Furthermore the software calculates the centrality and frequency of keywords where a higher keyword centrality value indicates greater influence (Chen, Zhang, et al. 2024; Chen, Cheng, et al. 2024). This study examined the top 20 high‐frequency keywords from the WOS and CNKI databases (Table 1).

**TABLE 1:** The top 20 high‐frequency keywords in CNKI and WOS literature on karst microhabitats research.

CNKI					WOS				
Number	Keywords	Centrality	Count	Year	Number	Keywords	Centrality	Count	Year
1	Karst	0.83	59	1998	1	Diversity	0.14	155	2002
2	Microhabitat	0.30	38	1995	2	Biodiversity	0.08	114	2002
3	Habitat	0.17	14	2003	3	Evolution	0.16	87	1991
4	Ecological niche	0.07	13	2004	4	Conservation	0.17	87	2001
5	Rocky desertification	0.11	9	2005	5	Community	0.10	77	1991
6	Microclimate	0.02	8	1995	6	Patterns	0.11	69	1991
7	Maolan	0.07	7	2003	7	Ecology	0.09	67	2002
8	*Drepanostachyum luodianense*	0.02	6	2008	8	Habitat	0.08	50	2007
9	Bryophytes	0.05	6	1998	9	Climate change	0.10	49	2005
10	Vegetation restoration	0.03	5	2007	10	Cave	0.04	45	2009
11	Drought stress	0.04	5	2011	11	Assemblages	0.03	39	2002
12	Karst region	0.12	5	1999	12	Karst	0.04	38	2000
13	Biomass	0.04	4	1998	13	Water	0.04	36	2009
14	Photosynthesis	0.03	4	2011	14	Vegetation	0.02	36	1994
15	Huajiang	0.03	4	2006	15	Forest	0.02	34	2014
16	Vegetation type	0.01	4	2010	16	Populations	0.07	34	1991
17	Ecological restoration	0.03	4	2007	17	New species	0.01	32	2009
18	Guizhou	0.01	4	2005	18	Species richness	0.03	27	2003
19	Leaf area	0.01	4	2012	19	Fauna	0.03	27	1996
20	Organic carbon	0.02	3	2010	20	Dynamics	0.06	27	2011

*Note:* A higher centrality value indicates greater influence of the keyword within the network.

Literature in both CNKI and WOS databases includes shared keywords such as “Karst”, “Habitat”, and “Vegetation”. However, the CNKI literature predominantly focuses on rocky desertification control and ecological restoration (Peng, Tang, et al. 2023; Peng, Xu, et al. 2023), highlighting that domestic research is largely driven by the need to address severe ecological degradation in Karst regions. In contrast, the WOS literature shows greater emphasis on biodiversity and community assembly patterns within microhabitats (Breg et al. 2018), reflecting an international research priority aimed at understanding theoretical ecological issues such as species diversity distribution, community assembly mechanisms, and adaptive evolution in karst microhabitats.

#### 2.3.4 Keyword Cluster Analysis

**FIGURE 3:**
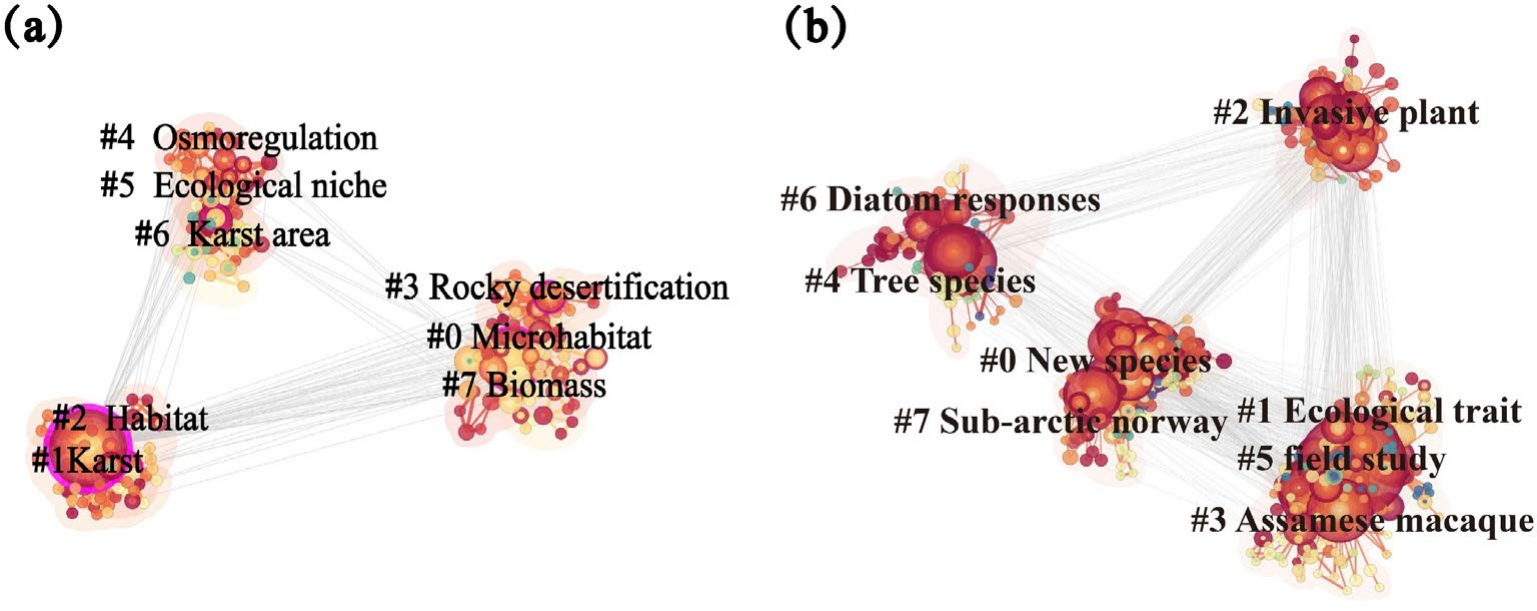
(a) CNKI database microhabitat keyword cluster; (b) WOS database microhabitat keywords cluster; larger nodes indicate clusters of high‐frequency keywords.

Keyword clustering reflects the main research directions of the selected literature and outlines the overall framework of microhabitat research (Figure 3). This study employed the log‐likelihood ratio (LLR) method to analyze keyword co‐occurrence mapping for clustering using the *Q*‐value and *S*‐value to evaluate clustering effectiveness. A clustering structure was considered significant when *Q* > 0.3 and results were deemed reasonable when *S* > 0.5 (Sood et al. 2022). Software calculations revealed modularity indices of *Q* = 0.71 and *S* = 0.91 for CNKI and *Q* = 0.49 and *S* = 0.78 for WOS meeting the criteria of *Q* > 0.3 and *S* > 0.5 thus providing reliable reference values.

Figure 3 highlights the 10 keyword clusters central to research in the CNKI and WOS databases. We observed that the CNKI research clusters (#0 microhabitat, #1 Karst, #6 Karst area) exhibit a region‐oriented focus, primarily addressing specific geographical units and ecological environmental issues (Peng, Dai, et al. 2020; Peng, Song, et al. 2020). In contrast, the WOS clusters (#0 New species, #4 Tree species, # 1 Ecological trait) display a mechanism‐oriented approach, concentrating on specific taxa, such as cave organisms and tree diversity along with their classification, functional traits, and adaptive mechanisms (Cardoso et al. 2022; Vilhar et al. 2022). Overall, while CNKI and WOS keyword clusters reflected field‐specific research hotspots, their distinct and non‐overlapping clustering patterns may hinder academic exchange due to differing research directions.

## 3 Overview of the Basic Characteristics of the Microhabitat

### 3.1 Terrestrial Microhabitat

**FIGURE 4:**
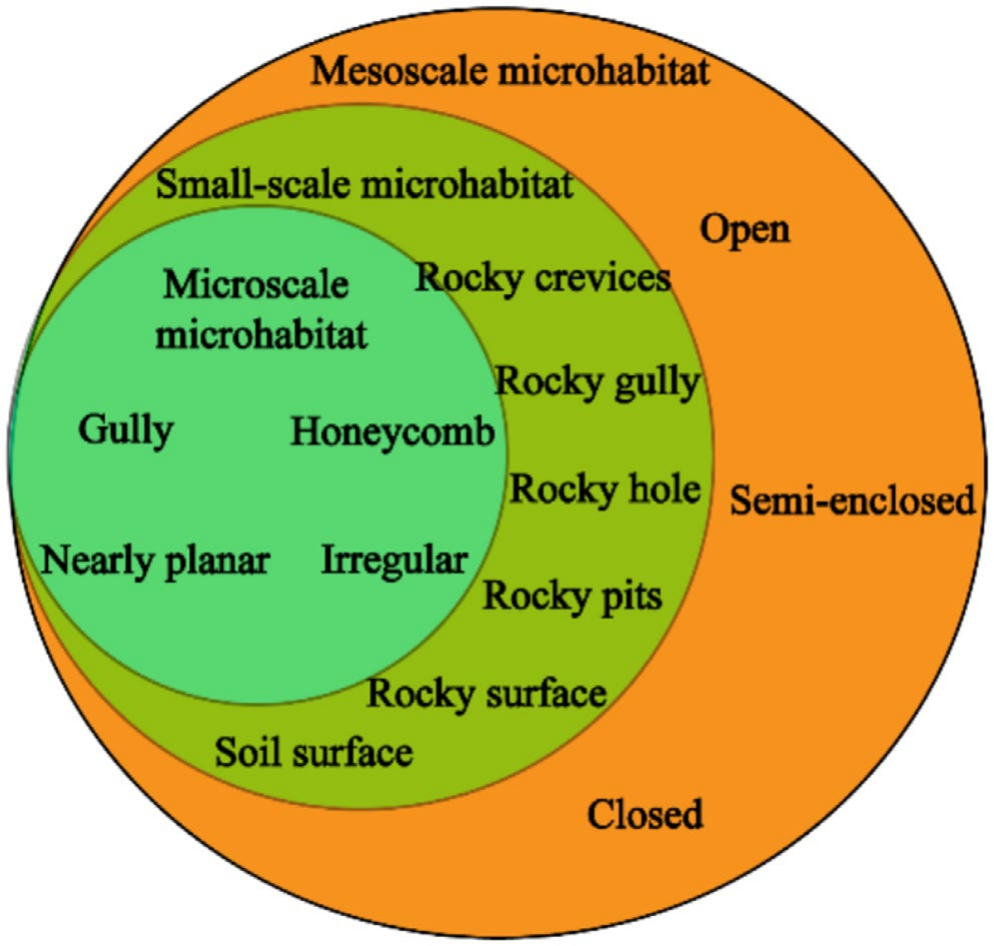
Surface microhabitat structure in karst area. (Green denotes microscale microhabitats, light green denotes small‐scale microhabitats, and orange denotes mesoscale microhabitats.)

Studies on the characteristics of aboveground microhabitats are often linked to the organisms growing on their surface. As biological species differ and habitats vary in scale, the scale of microhabitats is relative and lacks a unified standard (Zhu et al. 2021). We categorized microhabitats into microscale microhabitat, small‐scale microhabitat, and mesoscale microhabitats based on their distinct structural manifestations, with each scale exhibiting unique morphological features while maintaining ecological interconnections (Figure 4).

#### 3.1.1 Microscale Microhabitat

**FIGURE 5:**
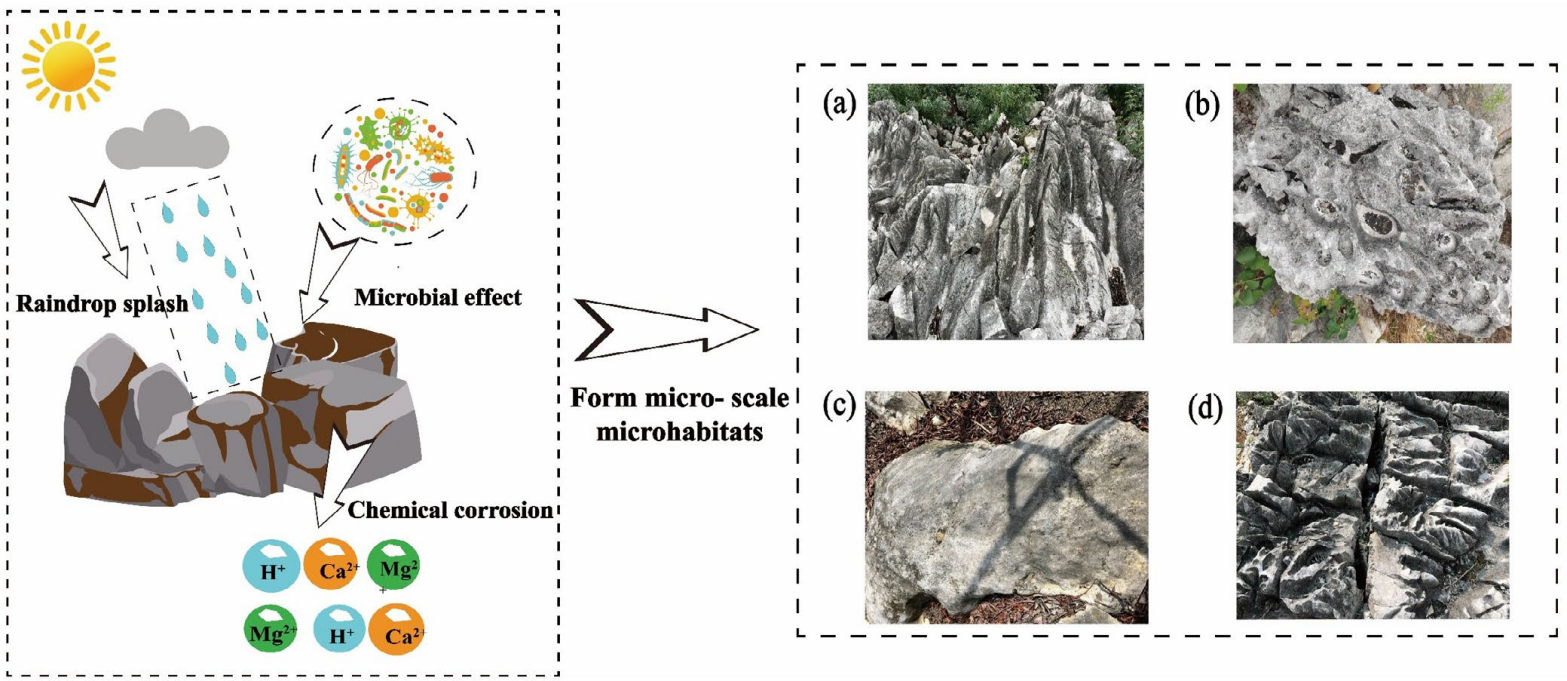
Microscale microhabitats in karst area. (a) Gully microhabitat; (b) Honeycomb microhabitat; (c) Nearly planar microhabitat; (d) Irregular microhabitat.

Microscale microhabitats serve as the fundamental functional units in the epigean ecosystems of Karst regions in China. Their formation results from differential dissolution of soluble rocks, creating typical morphologies such as grooved, honeycombed, irregular, and near‐planar surfaces (Figure 5). Previous studies have preliminarily characterized these microhabitats (Gong and Huang 1984; Mahoney et al. 2018) and identified the influence of underlying factors such as lithology, hydrology, and vegetation on their development.

However, the current research paradigm focusing on Chinese Karst regions exhibits certain limitations. There is a lack of quantitative classification standards for microscale microhabitat morphology, hindering objective comparison and integration of findings across different regions or even within the same area. Furthermore, while numerous studies have focused on the spatial heterogeneity of soil nutrients associated with microscale microhabitats (Waltham 2008; Cen et al. 2023), less attention has been paid to how the morphology of these microhabitats drives ecological processes by altering local hydrothermal conditions and influencing biological colonization.

Recent research has begun to reveal the complexity of these mechanisms. For instance, in rocky desertification restoration practices in China, pioneer organisms such as mosses colonize rock surfaces and subsequently modify the rock substrate through bio‐weathering and pedogenesis, acting as key drivers of microscale microhabitat evolution (Jiang et al. 2023). Similarly, Zerga (2024) highlighted the hydrogeological cycle as a critical “engine” in the formation of microscale microhabitats during karst landscape evolution. Nevertheless, a systematic and mechanistic understanding of the coupling between microhabitat morphology and ecological function remains elusive. Future studies should integrate geomorphometry, micro‐environmental monitoring, and controlled experiments to unravel the causal linkages among form, process, and function, thereby bridging the knowledge gap from microscale structure to macro‐scale ecological effects.

#### 3.1.2 Small‐Scale Microhabitat

**FIGURE 6:**
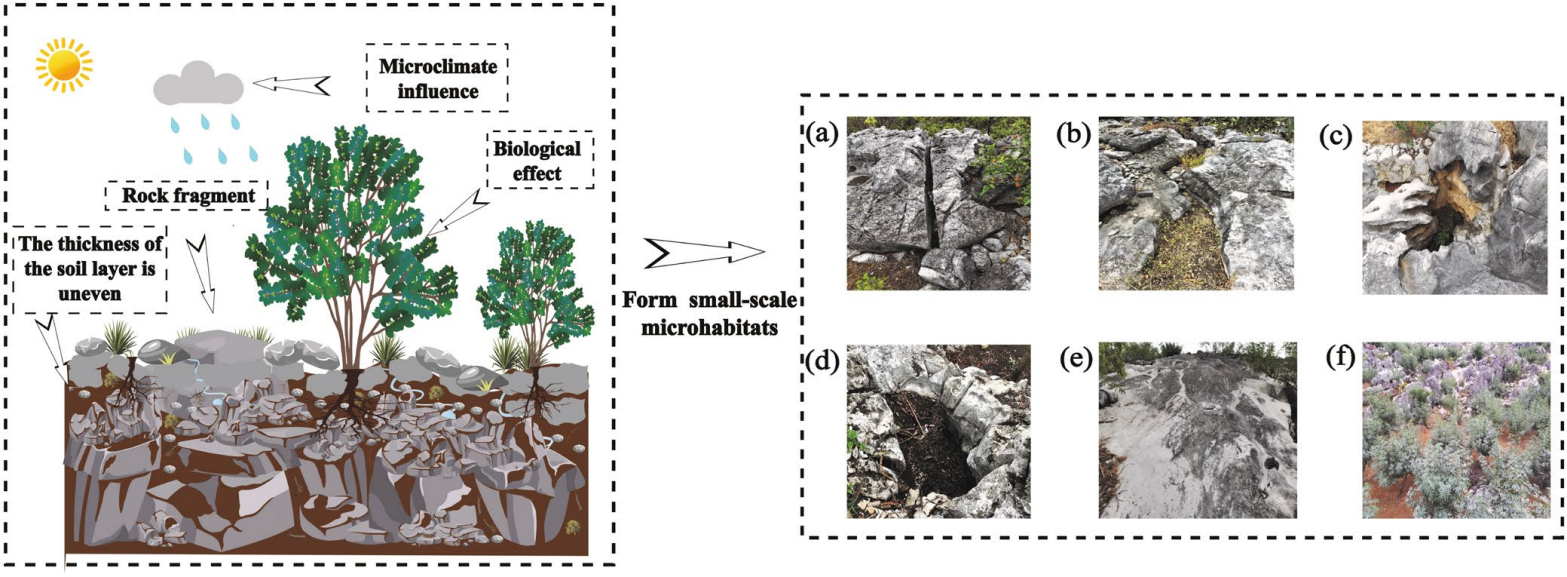
Small‐scale microhabitats in karst area. (a) Rocky crevices microhabitat; (b) Rocky gully microhabitat; (c) Rocky hole microhabitat; (d) Rocky pits microhabitat; (e) Rocky surface microhabitat; (f) Soil surface microhabitat.

The unique geological context of the karst region in southwestern China, characterized by high bedrock exposure, complex microtopography, and patchy vegetation, has given rise to diverse small‐scale microhabitats such as rock fissures, grooves, cavities, pits, exposed bedrock, and soil surfaces (Liu et al. 2008; Figure 6). Due to the compositional heterogeneity of these microhabitats, the karst system represents a typically heterogeneous ecosystem. Hamm and Drossel (2017) suggested that heterogeneous microhabitats can support biodiversity by providing broader niche space, a conclusion consistent with findings by Yang et al. (2021) in the same region. Although existing studies have systematically classified these microhabitats based on morphology, the key ecological processes within them and the mechanisms driving adaptive biological differentiation remain poorly understood.

Regarding biological responses, researchers have identified a range of adaptive strategies in karst‐endemic plants, particularly in organ traits and hydraulic architecture (Choat et al. 2018; Min et al. 2020). These findings provide a critical foundation for understanding plant survival strategies in karst environments and have revealed trait combinations geared towards transpiration reduction and optimized nutrient storage (Zhong et al. 2018). However, it remains unclear how microhabitat heterogeneity and shifts in species diversity drive the reorganization of leaf trait networks in degraded karst forests. Current studies largely focus on aboveground responses, while the mechanisms underlying root sensing and foraging in highly patchy resource environments remain a “black box.” Most hypotheses remain conceptual, lacking direct evidence from in situ observations or experiments. Future studies must link root foraging plasticity to the spatio‐temporal heterogeneity of soil resources. This integration is critical for unraveling the complete adaptation mechanisms of karst plants and for enhancing the predictive accuracy of ecosystem responses under global change.

#### 3.1.3 Mesoscale Microhabitat

**FIGURE 7:**
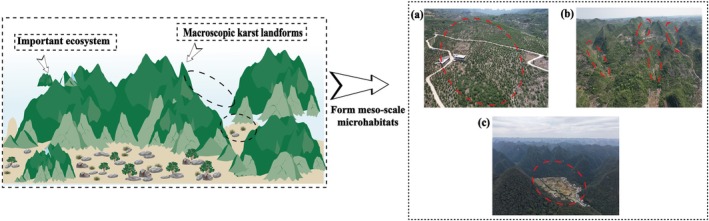
Mesoscale microhabitats (a) Open microhabitat; (b) Semi‐enclosed microhabitat; (c) Enclosed microhabitat.

Karst landscapes are both large‐scale land units and significant ecosystems (Domazetović et al. 2024). In the karst regions of southern China, mesoscale microhabitats are defined by macro‐karst landforms and classified into open, semi‐enclosed, and enclosed types based on topographic closure; examples include tiankengs, depressions, and peak clusters (Figure 7). These microhabitats play a critical role in maintaining biodiversity, yet their structural features also render them ecologically vulnerable (Bátori et al. 2017). Current domestic research on mesoscale microhabitats focuses mainly on element cycling and species diversity (Yang et al. 2025; Wang et al. 2025), while international studies more often address their formation mechanisms (Cahalan and Milewski 2018), highlighting a clear geographic divergence in research emphasis.

In ecological research, Huang et al. (2024) and Wang et al. (2022) applied Bayesian network models and benefit transfer methods, respectively, to assess ecosystem services in the karst regions of southwestern China. While these approaches introduced methodological innovations, their assessments still suffer from strong subjectivity and high model uncertainty. There is a need to develop more objective and scientifically robust ecological models that integrate multi‐source data to improve assessment accuracy. Moreover, although previous studies have highlighted the unique role of mesoscale microhabitats in resource allocation, microbial diversity, and karst carbon sink potential (Ojeda et al. 2019; Huang et al. 2023), the underlying mechanisms remain poorly integrated into a unified theoretical framework. For instance, systematic understanding of how bedrock geochemistry regulates vegetation growth, and how human activities and climate change jointly drive karst carbon sinks, is still lacking, particularly in cross‐regional and multi‐scale comparative analyses. Future research should place case studies from southwestern China's karst regions within a global karst research framework to enhance the applicability and generalizability of findings.

### 3.2 Subterranean Microhabitat

The karst ecological environment is fragile, having formed a surface‐subsurface dual geological structure through long‐term chemical dissolution (Balestra et al. 2024). We categorize the subterranean karst microhabitats of southern China into two types: shallow fissure‐porosity microhabitats, which are strongly influenced by the external environment, and the more environmentally isolated deep cave microhabitats.

#### 3.2.1 Shallow Fissure and Pore Microhabitat

Southwest China comprises one of the world's largest contiguous karst regions (Hartmann et al. 2014). Prolonged dissolution has developed extensive fissures, forming a distinct surface‐subsurface hydrological duality (Khoury et al. 2023). Unlike Mediterranean karst areas, its steep slopes and rugged topography, largely exceeding a 20% gradient, result from multiple tectonic events and provide substantial energy for surface runoff (Jiang et al. 2014). Driven by complex geology, heterogeneous landscapes, unsustainable land use, and abundant rainfall, approximately 29% of this region experiences rocky desertification and severe soil erosion (Zhang et al. 2014).

Although existing studies offer detailed descriptions of fissure geometry (Yan, Dai, Jin, et al. 2019; Yan, Dai, Wang, et al. 2019), the linkage between morphological classifications and hydro‐ecological functions remains poorly quantified. This gap limits our ability to predict the movement of water and solutes within fissure networks. Notably, Yan, Dai, Jin, et al. (2019); Yan, Dai, Wang, et al. (2019) observed trees growing on bedrock surfaces while shrubs dominated adjacent fissures, suggesting a specialized role of fissure microhabitats in water redistribution and root competition. Therefore, there is a critical need to integrate fissure distribution, morphological characteristics, and regolith profile data to clarify the sources and processes of soil infilling in karst fissures and to estimate infilling rates. This will improve the theoretical foundation for soil erosion prediction. Future studies should emphasize cross‐regional and cross‐lithological comparisons to identify the key factors controlling fissure development and function.

#### 3.2.2 Deep Cave Microhabitat

Deep caves formed by dissolution, characterized by perpetual darkness, nutrient scarcity and stable microclimates, constitute unique subterranean microhabitats. These caves occur at an average density of 6.4 per/km^2^ in Chinese karst regions (Ren et al. 2021; Hajna et al. 2024). These environments serve as natural laboratories for studying adaptive evolution and specialized ecological relationships (Deharveng and Bedos 2018). However, current research on cave biota demonstrates significant geographical and taxonomic biases. Most faunal studies focus on macroscopic animals including the Mexican cavefish ( *Astyanax mexicanus* ; Imarazene et al. 2021), horseshoe bats (*Rhinolophus* spp.; Straka et al. 2020), and millipedes (*Glyphiulus* spp.; Zhao et al. 2022), emphasizing their dependence on external nutrient inputs.

In contrast, high‐throughput sequencing has progressively revealed the diversity and ecological functions of microbial communities driving cave material cycling (Havlena et al. 2021). Different cave systems exhibit high heterogeneity in both microhabitat structure and biological communities. In contrast to the cave vascular flora in Sicilian karst, which was dominated by bryophytes and ferns (Puglisi et al. 2019), the flora documented in Chinese karst caves was dominated by angiosperms (Monro et al. 2018). This discrepancy may be attributed to differences in regional climate, karst geomorphology, and levels of anthropogenic disturbance. Recent research by Webster et al. (2022) proposes caves as potential methane sinks, closely associated with the diversity, distribution and interactions of methanotrophic bacteria. This perspective offers new insights into understanding caves' roles in biogeochemical cycles. Future studies should focus on elucidating the distribution of microbial functional genes and interaction networks to better evaluate the potential functions of cave ecosystems.

## 4 Utilization of Microhabitats

### 4.1 Water and Fertilizer Management and Agricultural Production

Water and nutrient are crucial elements for plant growth and development. Yet the distinctive “binary three‐dimensional” structure of karst regions in China poses significant challenges, including severe water and soil loss as well as ecological fragility. These constraints often render conventional water and fertilizer management strategies ineffective, limiting crop productivity (Liu et al. 2020; Fonseca et al. 2023). Developing management approaches that account for the specific characteristics of karst microhabitats is therefore crucial for enhancing agricultural productivity in these areas.

Effective management hinges on optimizing water use and fertilization practices. Regarding water use, to address geological and seasonal drought (Malagò et al. 2016), technologies like the “retention‐storage” water‐conservation system (Jiang et al. 2019), superabsorbent polymers (Hanikel et al. 2021), and canopy structure regulation (Wu and Wang 2024) have been proposed. However, evaluations of these technologies remain limited to short‐term effects and specific pilot sites, lacking long‐term efficacy and cost–benefit analyses across heterogeneous landscapes. This has resulted in a fragmented technological framework. Compared to the mature and standardized water‐saving agricultural systems in non‐karst regions of China, karst areas lack a systematic technical system based on the genesis and functional classification of microhabitats, underscoring the need for location‐specific management protocols.

Regarding fertilization, controlled‐release fertilizers offer a means to improve nutrient use efficiency while minimizing environmental impact (Yang et al. 2012). However, the widespread distribution of limestone soils in Chinese karst regions, characterized by high calcium and magnesium content and an alkaline pH, not only induces physiological drought but can also immobilize key elements such as phosphorus and zinc, reducing their availability (Dugan et al. 2024). Consequently, future water and nutrient management strategies in Chinese karst agriculture must evolve beyond simple water‐fertilizer coupling towards an integrated systemic approach that synergistically manages water, fertilizers, and soil. This integrated strategy should aim to enhance nutrient use efficiency through improved water management and optimize water utilization via tailored fertilization.

### 4.2 Ecological Adaptation and Management of Forest Trees

Tree spatial distribution reflects individual adaptive strategies to environmental conditions (Condit et al. 2000). Point pattern analysis offers distinct advantages in characterizing such spatial patterns (Qi et al. 2021). However, in the karst regions of southwestern China, where poor water retention and high soil nutrient leakage prevail (Li et al. 2025), effective vegetation restoration depends on precise microhabitat identification and species‐habitat matching.

Selecting suitable tree species based on microhabitat characteristics is essential for establishing stable communities in karst ecological restoration. For example, Yu et al. (2018) implemented species–site matching in rocky desertification areas of Guizhou according to microhabitat features. While Zhao et al. (2023) utilized the deep‐rooted, fast‐colonizing species (*Alchornea trewioides*(*Benth*.) *Muell Arg*) as a pioneer plant in arid habitats. Still, a predictive framework linking plant functional traits to habitat factors remains underdeveloped. Community restoration follows nonlinear dynamics regulated by resource gradients, species strategies, and post‐disturbance conditions. Studies by Cheng et al. (2023) and Kermavnar et al. (2023) demonstrate how regeneration patterns shift with succession stages and microhabitat types, revealing inherent trade‐offs and path dependence during community assembly. Thus, successful restoration requires not only appropriate species selection but also proactive guidance of successional trajectories.

Although tree germination is species and genotype‐dependent, habitat heterogeneity modulates tree size, germination vigor, and neighbor competition—thereby influencing community assembly and regeneration (Qi et al. 2022). Future studies should leverage material fluxes across microhabitats to refine the visual topology of tree distributions and elucidate ecological drivers of community adaptation within the critical zone, ultimately enhancing the resilience of karst forest ecosystems in China.

### 4.3 Land Use and Ecological Restoration

The impact of land use on biodiversity and ecosystem functioning has become a key research topic globally (Asabere et al. 2020). In the fragile microhabitats of karst southwestern China, land use expansion can accelerate land degradation. This process is closely associated with vegetation and management practices that alter soil properties and aggregate stability in surface microhabitats (Gan et al. 2024). For instance, Li et al. (2015) demonstrated that young forest land and sloping farmland exhibit the most unstable soil structure and pose the greatest challenges for ecological restoration in Guizhou karst regions, highlighting the critical role of land use planning in rehabilitation efforts.

Current research is shifting from focusing solely on surface processes to integrating subsurface dynamics. Studies reveal that fissure microhabitats in karst regions are enriched with nutrients, where tree roots develop multi‐directional branching strategies to adapt to these unique conditions, thereby enhancing soil stabilization (Fu et al. 2022). This understanding has promoted a transition in restoration strategies from singular biological measures to systematically enhancing ecological functions through optimized land use structures. Supporting evidence comes from Brandt et al. (2018), who demonstrated that converting degraded lands to woodlands effectively improves habitat quality and mitigates degradation in south China's karst areas. However, methods for quantitatively incorporating subsurface processes, such as root reinforcement in fissures and preferential flow solute transport into restoration assessments remain underdeveloped.

In ecological source identification, early approaches often simplistically equated large, high‐quality habitat patches with source areas for species dispersal (Gao et al. 2022). Such methods tend to be subjective and inadequately account for dynamic landscape processes and habitat heterogeneity, thereby limiting restoration effectiveness. Moving forward, under global karst habitat change scenarios, integrating both surface and subsurface characteristics of microhabitats will be essential. This integration will facilitate a shift from managing individual ecological elements towards comprehensive ecosystem management, representing a necessary evolution for establishing effective ecological security patterns in southern China's karst regions.

## 5 Research Outlook

### 5.1 Expansion of Quantitative Microhabitat Research

Previous studies had verified that microhabitats are highly valuable for species survival due to their complex structures. However, current research on karst microhabitats in China remains largely qualitative in approach, and quantitative research is still in its preliminary stages. Future studies should employ the structure–function principle and morphometrics to quantify microhabitat morphology and their erosional environments. This will lay the groundwork for clarifying the interrelationships between microhabitat, soil nutrients, biomass, and species diversity.

### 5.2 Strengthening Microclimate Research

The complex topography of China's karst region creates diverse microhabitats with pronounced microclimatic heterogeneity, profoundly influencing species survival, agricultural productivity, and disaster mitigation. However, limited systematic observational data and insufficient pattern summarization have constrained our understanding of these microclimatic characteristics, hindering their practical application. Future research should enhance microclimate monitoring and analysis when studying karst ecosystem processes and functions. Moreover, when designing generalizable microclimate simulation experiments, habitat heterogeneity must be incorporated to ensure scientific validity and extrapolation potential.

### 5.3 Clarifying Patterns of Resource Allocation at Different Microhabitat Scales

Given the high degree of spatio‐temporal heterogeneity in the resource allocation of different microhabitats, it is challenging for plants to achieve balanced resource utilization. In the future, from a geographical perspective, we should build a spatio‐temporal model of resources and environmental elements in Chinese karst areas. This will help reveal the mechanism of nutrient and energy flow and distribution among microhabitats at different scales, providing a theoretical basis for the optimal allocation of habitat resources. Furthermore, it is essential to integrate the theory of the Earth's critical zone to uncover the resource allocation laws of small habitats at different scales. This is fundamental for the efficient utilization of resources.

### 5.4 Improving Surface‐Subsurface Microhabitat Connectivity Studies

**FIGURE 8:**
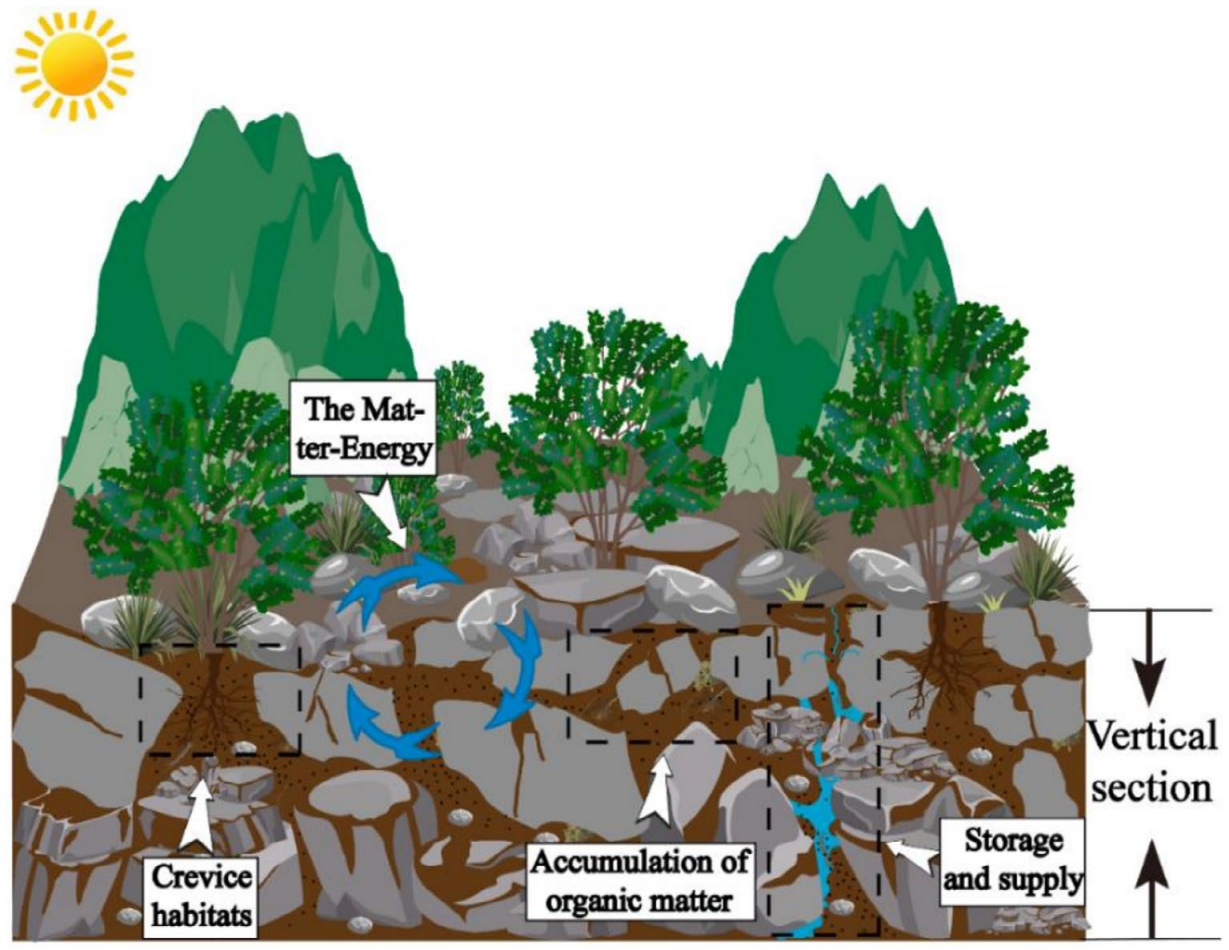
Connectivity mechanisms between surface and subsurface microhabitats in karst area.

The connectivity between surface and subsurface microhabitats complicates the pathways of solute transport and utilization. Understanding this relationship can enable the regulation of the transport and utilization of soluble material. Current modeling approaches primarily rely on geometric parameters to construct three‐dimensional fracture network models, which oversimplifies the complexity of karst fissure habitats in southern China and limits accurate simulation of water‐rock interactions and material transport processes. The theoretical perspective in traditional landscape ecology remains largely confined to two‐dimensional spatial analysis, lacking an integrated three‐dimensional consideration of vegetation‐soil‐subsurface ecosystems. Future research should characterize the structural and functional connectivity between surface and subsurface systems, and reveal their dynamic evolution based on engineering practices and water‐rock interaction patterns in typical Chinese karst regions (Figure 8).

## Author Contributions

**Hui Huang:** data curation (equal), formal analysis (equal), investigation (equal), methodology (equal), software (equal), validation (equal), visualization (equal), writing – original draft (equal). **Yanghua Yu:** conceptualization (equal), funding acquisition (equal), project administration (equal), supervision (equal), writing – review and editing (equal). **Shunsong Yang:** investigation (equal), validation (equal), visualization (equal). **Yun Yang:** investigation (equal). **Yurong Fu:** investigation (equal), validation (equal).

## Funding

This work was supported by the Science and Technology Program of Guizhou Province, Qian‐ke‐he Zhicheng [2023] Yiban 062.

## Ethics Statement

The authors have nothing to report.

## Conflicts of Interest

The authors declare no conflicts of interest.

## Data Availability

The data that support the findings of this study are openly available in Archive of OSF Storage at https://doi.org/10.17605/OSF.IO/S892Q.
